# Automatic Segmentation of Novel Coronavirus Pneumonia Lesions in CT Images Utilizing Deep-Supervised Ensemble Learning Network

**DOI:** 10.3389/fmed.2021.755309

**Published:** 2022-01-03

**Authors:** Yuanyuan Peng, Zixu Zhang, Hongbin Tu, Xiong Li

**Affiliations:** ^1^School of Electrical and Automation Engineering, East China Jiaotong University, Nanchang, China; ^2^School of Computer Science, Northwestern Polytechnical University, Xi'an, China; ^3^Technique Center, Hunan Great Wall Technology Information Co. Ltd., Changsha, China; ^4^School of Software, East China Jiaotong University, Nanchang, China

**Keywords:** under CT imaging, deep learning, COVID-19 lesion segmentation, deep-supervised ensemble learning network, transfer learning, local and global features

## Abstract

**Background:** The novel coronavirus disease 2019 (COVID-19) has been spread widely in the world, causing a huge threat to the living environment of people.

**Objective:** Under CT imaging, the structure features of COVID-19 lesions are complicated and varied greatly in different cases. To accurately locate COVID-19 lesions and assist doctors to make the best diagnosis and treatment plan, a deep-supervised ensemble learning network is presented for COVID-19 lesion segmentation in CT images.

**Methods:** Since a large number of COVID-19 CT images and the corresponding lesion annotations are difficult to obtain, a transfer learning strategy is employed to make up for the shortcoming and alleviate the overfitting problem. Based on the reality that traditional single deep learning framework is difficult to extract complicated and varied COVID-19 lesion features effectively that may cause some lesions to be undetected. To overcome the problem, a deep-supervised ensemble learning network is presented to combine with local and global features for COVID-19 lesion segmentation.

**Results:** The performance of the proposed method was validated in experiments with a publicly available dataset. Compared with manual annotations, the proposed method acquired a high intersection over union (IoU) of 0.7279 and a low Hausdorff distance (H) of 92.4604.

**Conclusion:** A deep-supervised ensemble learning network was presented for coronavirus pneumonia lesion segmentation in CT images. The effectiveness of the proposed method was verified by visual inspection and quantitative evaluation. Experimental results indicated that the proposed method has a good performance in COVID-19 lesion segmentation.

## Introduction

Since the end of 2019, acute infectious pneumonia characterized by novel coronavirus disease 2019 (COVID-19) infection has been rapidly spread in the world, posing a huge threat to the lives of people (1). The outbreak of pneumonia caused by COVID-19 infection has been identified by the WHO as an emergency public health event of international concern, the number of patients with COVID-19 is rapidly growing in the world (2). So far, the cumulative confirmed cases of COVID-19 in the world exceeded 200 million, and the cumulative deaths reached 3.6 million. The early symptoms of pneumonia are not obvious but are strongly infective. It has caused huge economic losses to society and aroused wide concern in the world (3, 4).

The difficulty of prevention and treatment of COVID-19 has put forward urgent requirements to the research of rapid diagnosis methods. The prevention and control measures of early diagnosis, early isolation, and early treatment for patients with COVID-19 are one of the most effective strategies to solve the pneumonia epidemic (5). However, widely used nucleic acid testing and specific antibody detection technologies have several disadvantages, such as lagging, long time consuming, low detection efficiency, and serious risk of missed detection (6). As one of the most effective lung imaging modes, CT has the ability to identify the changes in lung lesions and pathological features in patients with COVID-19 (7, 8). Therefore, a large number of researchers have designed many different deep learning models to assist clinic doctors in the rapid diagnosis of COVID-19 in CT images (9).

Traditional methods achieve the purpose of different semantic segmentation tasks by extracting features of the target objects (10–12), but the segmentation performance was not good enough. To overcome the problem, various deep learning frameworks have been proposed to effectively segment the target objects (13). It could be divided into four classifications, deep-supervised learning, semi-supervised learning, weakly supervised learning, and unsupervised learning approaches in COVID-19 lesion segmentation. Compared with traditional methods, the segmentation performance has been largely improved by deep learning networks (14–16).

Although deep learning frameworks can achieve a good performance in COVID-19 lesion segmentation, they required a lot of data and the corresponding data annotations (17). To solve the problem, data augmentation is one of the most common operations, which can generate large amount of data through rotation, scaling, clipping, and transposing. However, using only data augmentation may cause some lesions to be undetected. Therefore, many advanced strategies, such as transfer learning, multi-task approach, and attention mechanism have been proposed to improve the performance of COVID-19 lesion segmentation. Based on this theory, a multi-task approach was designed by Yazdekhasty et al. to reach the purpose (18), it had a good performance in lacking data and model generalization. Using a different strategy, Wang et al. integrated with transfer learning, UNet model, and multi-task learning to improve the segmentation performance of COVID-19 lesions (19). Recently, an attention mechanism (19, 20) was employed to make up for the shortcoming of partial information missed caused by convolution operation in deep learning networks. To further improve the segmentation performance, semi-supervised learning strategies were proposed to train mounts of pseudo annotations. Based on this strategy, Zhao et al. presented a randomly selected propagation strategy to improve the segmentation performance of COVID-19 lesions (21). Similarly, Abdel-Basset et al. proposed an innovative semi-supervised few-shot segmentation method for COVID-19 lesion segmentation from a few amounts of annotated lung CT images (22). Existing supervised and semi-supervised methods require mounts of voxel-based annotations in the training stage (23). Unfortunately, it is difficult for clinicians to precisely annotate COVID-19 lesions due to the complex structural changes and blurred boundary information (24). To overcome this problem, mounts of weakly supervised methods have been proposed to segment COVID-19 lesions. The advantage of the weakly supervised approaches is that it can replace the complicated COVID-19 lesion labels with simple ones for training. Based on this strategy, Yang et al. presented a weakly supervised method based on a generative adversarial network (GAN) to improve the accuracy of COVID-19 lesion segmentation (25). A generator was adopted to remove lesions and generate healthy slices from input images, while a discriminator was used to force the generator to generate more accurate results with mounts of image-level annotations. The method was improved by Laradji et al. (26), where they utilized two encoder-decoder frameworks with shared weights. The first one encoded the original images, and the point annotations were treated as the corresponding supervised term. While the second one encoded the original image with geometric transformation, and the outputs of the first one with geometric transformation were regarded as the corresponding supervised term. Similarly, Wu et al. proposed a new 3D active learning framework called COVID-AL to segment COVID-19 lesions with volume-annotations (27). Recently, Wang et al. proposed a weakly supervised deep learning framework for COVID-19 lesion segmentation (28). First, a pre-trained UNet is applied to remove unrelated tissues for lung segmentation. Subsequently, the segmented lung is fed into the designed DeCoVNet to acquire the COVID-19 lesion feature map. Finally, a class activation mapping algorithm and a 3D connected component algorithm were combined for COVID-19 lesion localization. The fatal flaw of the deep supervised approach, semi-supervised approach, and weakly supervised approach is that mounts of data labels are required to supervise the training model in the training stage. Whereas data annotations need clinical experts to spend a lot of time to annotate it. Different from the above deep learning approaches, the unsupervised approach has a good performance in objects segmentation without any annotated labels. To alleviate the burden of data annotation, Yao et al. designed an unsupervised NormNet model to distinguish COVID-19 lesions from complex lung tissues (29). Based on the observation that parts of tracheae and vessels exhibit strong patterns, a three-stage (random shape, noise generation, and image filtering) strategy was used to generate lesion shape for subsequent segmentation. Taking the difference between COVID-19 lesions and other tissues into consideration, a novel method named NormNet based on generative adversarial networks was presented for COVID-19 lesion segmentation. Unfortunately, the unsupervised NormNet model had a bad performance than some supervised methods. The advantages and disadvantages of different types of deep learning methods are summarized in Table 1.

**Table 1:** Coronavirus disease 2019 (COVID-19) lesion segmentation with different methods.

	**Methods**	**Advantages**	**Disadvantages**
Deep-supervised learning	Yazdekhasty et al. (18)	Good performance in sufficient data and model generalization	Mounts of voxel-based Data annotation
	Wang et al. (19)		
	Gao et al. (20)		
Semi-supervised learning	Zhao et al. (21)	Good performance in lacking data	Parts of voxel-based Data annotation
	Abdel-Basset et al. (22)		
Weakly supervised learning	Yang et al. (25)	Class annotation	Poor segmentation in small lesions
	Laradji et al. (26)		
	Wu et al. (27)		
	Wang et al. (28)		
Unsupervised learning	Yao et al. (29)	No data annotations	Bad performance

Motivated by the fact that different deep learning methods have their own unique advantages, these advantages can be fused using a deep-supervised ensemble learning network to improve the COVID-19 lesion segmentation results in CT images. Unfortunately, the specific deep learning framework may take up more time and space.

In this study, a deep-supervised ensemble learning network is proposed for COVID-19 lesion segmentation in CT images. To alleviate the overfitting problem on small datasets, a transfer learning strategy is used to acquire initialization parameters with better feature performance. Subsequently, an enumeration grid model is exploited to estimate the optimal weights for multiple deep learning model integration. In particular, we pay special attention to the COVID-19 lesion and its boundary segmentation, which can illustrate the effectiveness of the proposed method. Compared with several methods, the proposed model has a good performance in COVID-19 lesion segmentation in CT images.

## Materials and Methods

In this study, we presented a deep-supervised ensemble learning network as an alternative model to segment COVID-19 lesions compared with several models in publicly available datasets. Visual inspection and quantitative evaluation were established in this study to verify the proposed ensemble learning network.

### Evaluation Criteria

**Figure 1:**
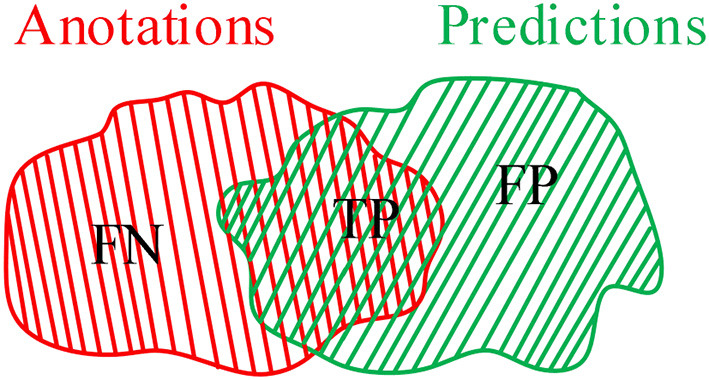
An intersection over union (IoU) criterion.

To illustrate the validation of the proposed method, an IoU criterion and *F*_1_-measure (10) are applied to verify the good performance. As shown in Figure 1, annotations A are divided into false negative (FN) and true positive (TP), whereas predictions B are divided into TP and false positive (FP). In which, TP is the common region between annotations A and predictions B. Therefore, the mathematical description of IoU, precision (P), recall (R), and *F*_1_ can be defined as follows:


(1) $$\begin{array}{r} IoU=\frac{TP}{TP+FP+FN} \end{array}$$

(2) $$\begin{array}{r} P=\frac{TP}{TP+FP} \end{array}$$

(3) $$\begin{array}{r} R=\frac{TP}{TP+FN} \end{array}$$

(4) $$\begin{array}{r} F_{1}=\frac{2\times P\times R}{P+R} \end{array}$$

In other words, the greater proportion of the common region between annotations and predictions, the greater IoU and *F*_1_ values will be. The smaller proportion of common region between annotations and predictions, the smaller IoU and *F*_1_ values will be. Especially in the case with small COVID-19 lesions, IoU has a strong ability to illustrate the effectiveness of the proposed method. Additionally, the *F*_1_-measure criterion denotes the similarity between annotations and predictions.

As we all know that IoU and *F*_1_ pay more attention to the regional sensitivity of image segmentation, but the description of the segmentation boundary is also important. To further illustrate the effectiveness of the proposed method, Hausdorff distance is employed to evaluate the validation of the deep learning framework. The Hausdorff distance from annotations A to predictions B can be defined as


(5) $$\begin{array}{r} h\left(A,B\right)=\underset{a\in A}{\max}\left\{\underset{b\in B}{\min}\left\{d\left(a,b\right)\right\}\right\} \end{array}$$

Where *h*(*A, B*) is the Hausdorff distance from A to B, *d*(*a, b*) is the distance between point a and point b. In which, point a and b are belonging to A and B, respectively. To illustrate the distance between A and B, a more general definition would be:


(6) $$\begin{array}{r} H\left(A,B\right)=\max\left\{h\left(A,B\right),h\left(B,A\right)\right\} \end{array}$$

**Figure 2:**
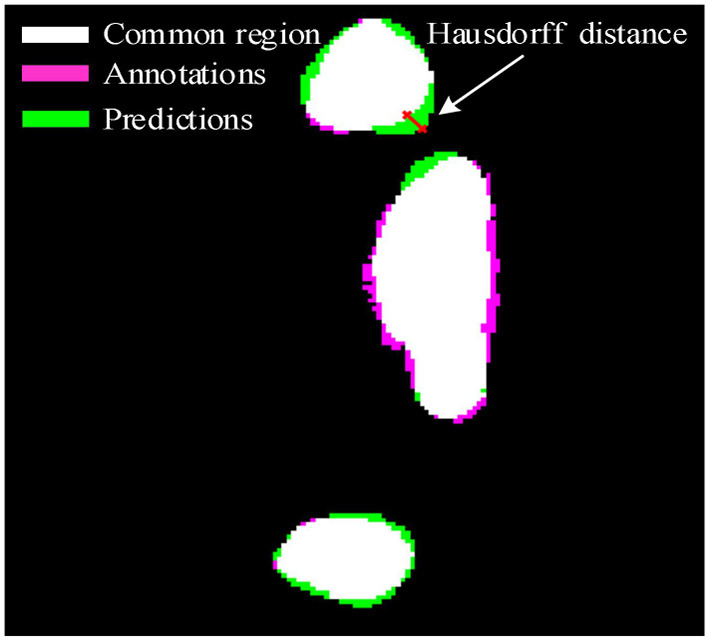
Hausdorff distance.

As shown in Figure 2, the pink area is the annotations A, the green area is the predictions B, and the white area is the common region covered by both predictions A and annotations B. Hausdorff distance measures the biggest distance from both predictions A and annotations B to common region. To avoid the influence of noise on Hausdorff distance, 95% Hausdorff distance (HD95) are treated as useful values sorted from small to large. To avoid Hausdorff distance being too large, a simple mathematical transformation is given as:


(7) $$\begin{array}{r} H=\overset{n}{\underset{i=1}{\sum }}\mathrm{lg}\left(HD95_{i}+1\right) \end{array}$$

Where *i* is the number of the test CT slices. In other words, the better detection of the COVID-19 lesion boundary, the smaller *H* value will be. Whereas the worse detection of the COVID-19 lesion boundary, the larger *H* value will be. The evaluation criteria IoU, *F*_1_, and *H* are used to evaluate the validation of the proposed method.

### Data and Annotations

We employed a dataset from the China consortium of chest CT image investigation (30), which is a publicly available dataset. The dataset includes 150 CT scans. In which, 750 CT slices were selected from the dataset and annotated by four doctors with extensive clinical experience, 400 CT slices were treated as a training set, 200 CT slices were considered as a validation set, and 150 CT slices were used as a test set. In this work, we treated the corresponding annotations as the ground truth.

### Overview of the Proposed Method

**Figure 3:**
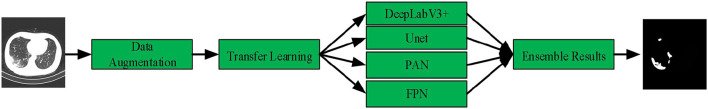
A pipeline for COVID-19 lesion segmentation in CT images.

In this article, we present a deep-supervised ensemble learning network for COVID-19 lesion segmentation in CT images in Figure 3. First, data augmentation is applied to increase the training data and improve the generalization ability of the model. Subsequently, a transfer learning strategy is employed to copy with small datasets and alleviate the overfitting problem. Finally, a deep-supervised ensemble learning network is presented to combine with local and global features for COVID-19 lesion segmentation in CT images.

### Data Augmentation

Clinicians spend a great deal of time to annotate complex structures of COVID-19 lesions, which is too expensive. In general, data augmentation (31) is applied to process and increase the training data to make the data as diverse as possible and make the training model generalization ability stronger. To reach the purpose, CT image operations, such as horizontal flip, vertical flip, rotation, and scaling are adopted to increase training data and the corresponding COVID-19 lesion annotations. After data augmentation, the number of training data is varied from 400 to 4,000.

### Transfer Learning

Deep learning models have been widely applied in medical image processing (32). To acquire accurate COVID-19 lesion segmentation results, a large amount of CT images and manual annotations are required to adjust the parameters for the special deep learning model. However, it is too difficult to obtain mounts of CT images and the corresponding manual lesion annotations. In general, the designed deep learning models are trained with small datasets, which may lead to poor generalization ability and serious overfitting performance. To cope with the shortcomings, many technical means, such as data augmentation (31), multi-task learning (33), transfer learning (34), and attention mechanism (35) can be used to achieve the segmentation task. In this section, a transfer learning strategy is applied to solve the shortcomings.

It is a challenging task for doctors to manually annotate complex and variable COVID-19 lesions. To acquire the best parameters of the designed model with the limited COVID-19 lesion annotations, a transfer learning strategy is employed to accurately segment COVID-19 lesions in CT images. In this, the ImageNet dataset is treated as the pre-training dataset, which is one of the largest image datasets in the world (36). Whereas the EfficientNet model (37, 38) is considered as the pre-training model. First, the model is trained with the training data and the corresponding manual annotations to generate pseudo annotations, it was treated as the teacher in the “teacher-student” model. Whereas a powerful EfficientNet model is retrained by using manual and pseudo annotations, it was considered as the student in the “teacher-student” model. In the student learning stage, adding noise processing is used to make the generalization ability of students better than teachers (39). In this section, the estimated parameters are regarded as the initialized parameters for the deep-supervised ensemble learning network.

### Deep-Supervised Ensemble Learning Network

Different networks have different advantages and disadvantages, the advantages can be integrated together by effectively integrating various networks. To acquire the best COVID-19 lesions segmentation performance, a deep-supervised ensemble learning network is presented for COVID-19 segmentation in CT images.

**Figure 4:**
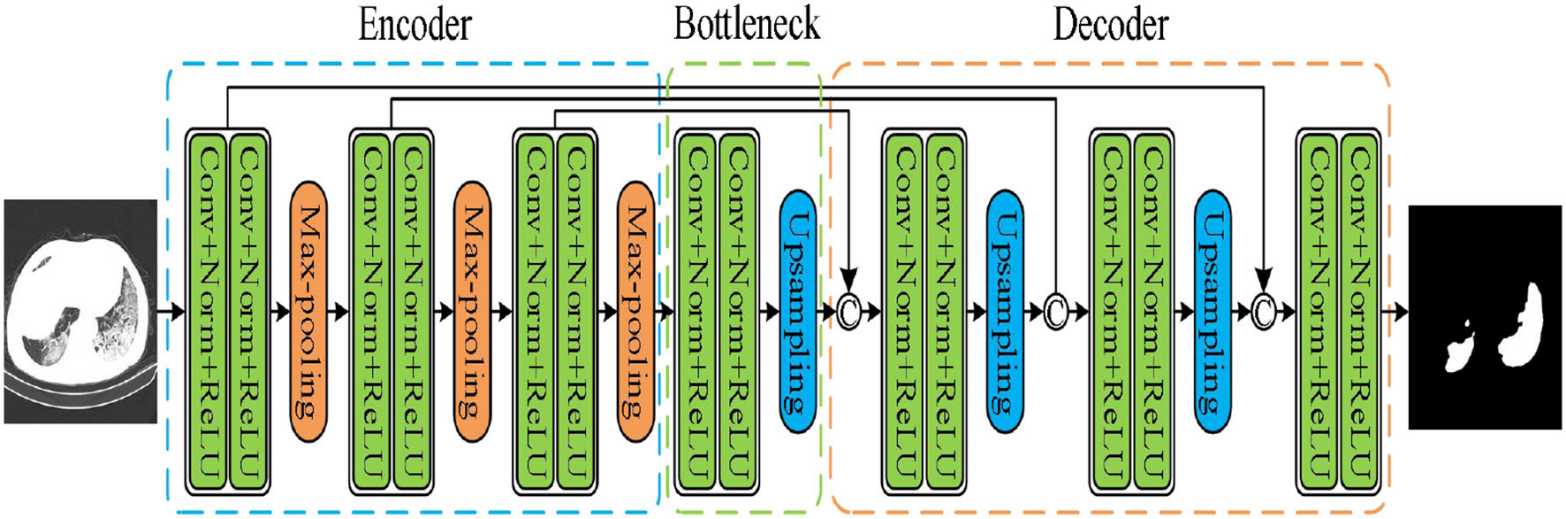
UNet model.

The UNet model was applied according to Su et al. and Wang et al. (40, 41). As shown in Figure 4, the network had three encoding and decoding blocks, respectively. Encoding was designed using max-pooling, whereas the decoding was performed *via* a deconvolution. In addition, the encoder and the decoder were connected *via* skip connections.

**Figure 5:**
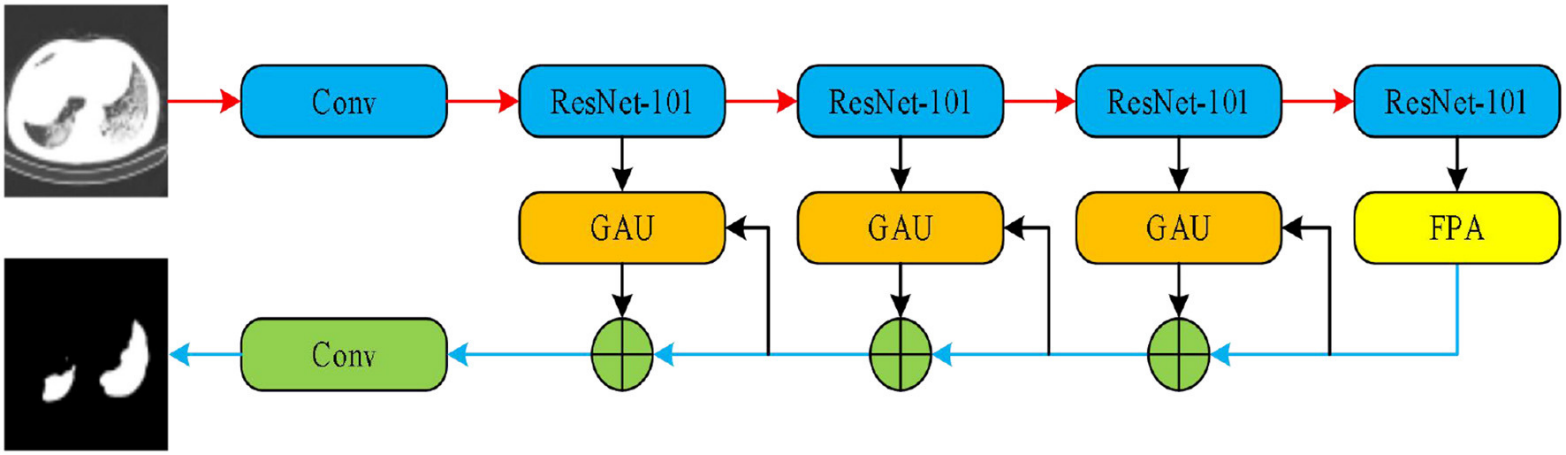
Pyramid attention network (PAN).

As a second architecture, we implemented the pyramid attention network (PAN) by Li et al. (42). Unlike the traditional deep learning model, the basic principle of the pyramid attention network is to effectively extract local and global features of target objects by integrating the attention mechanism and spatial pyramid structures. As shown in Figure 5, an encoder-decoder scheme was adopted to locate target objects. In the encoder module, a feature pyramid attention (FPA) was introduced to the adopted spatial pyramid attention mechanism in the high-level output, and the global information was applied to learn stronger feature representation. In the decoder module, a global attention upsample (GAU) module was applied to extract the global information of the target objects to effectively segment COVID-19 lesions.

**Figure 6:**
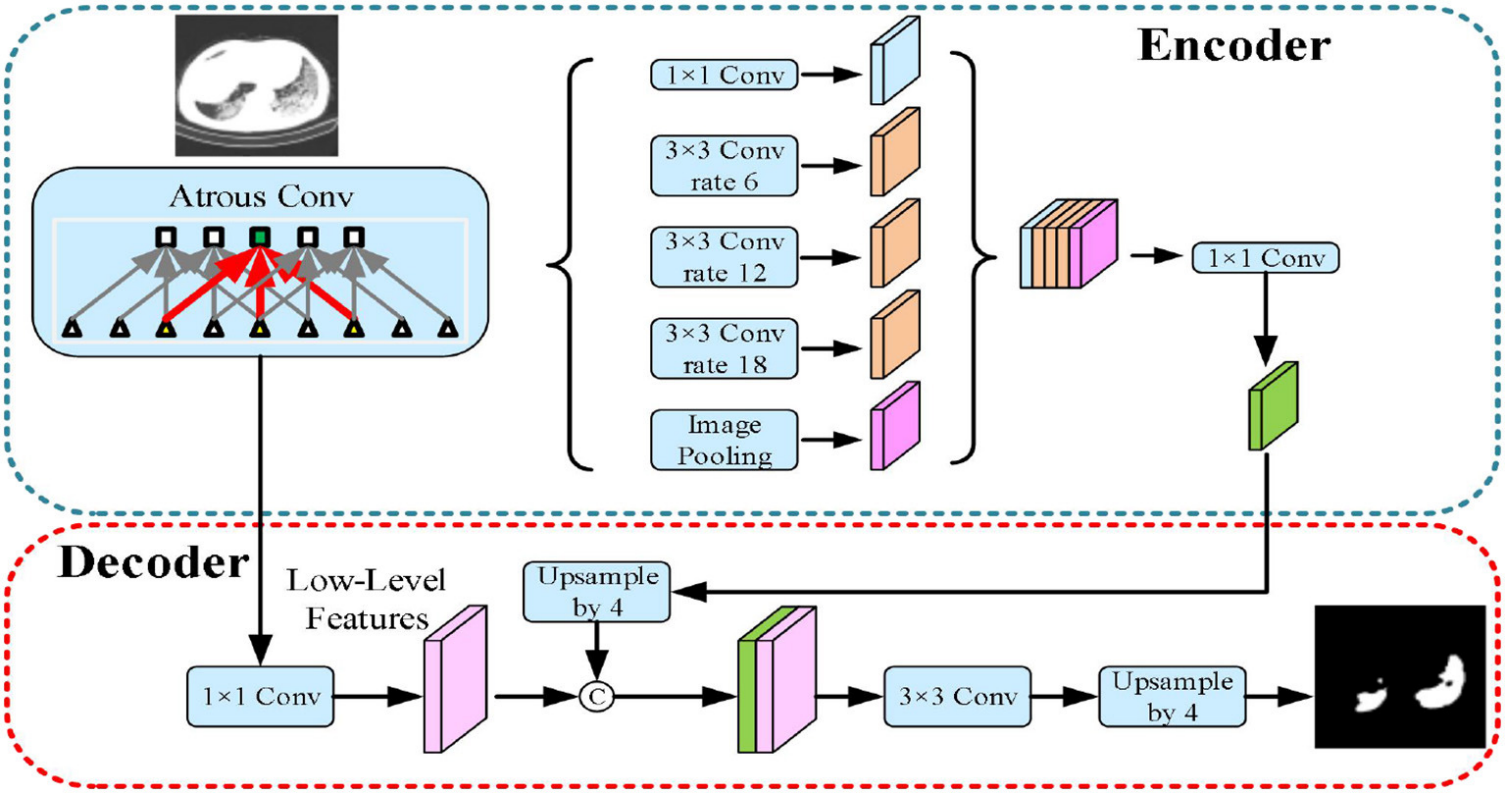
DeepLabv3+ model.

The third implemented architecture is the DeepLabv3+ by Chen et al. (43). As shown in Figure 6, it is the DeepLabv3 extended version by adding a decoder module to refine the segmentation results, especially along the novel coronavirus pneumonia lesion boundaries. Specifically, the Xception model was explored and the depthwise separable convolution was applied to both atrous spatial pyramid pooling and decoder modules, resulting in a faster and stronger encoder-decoder network.

**Figure 7:**
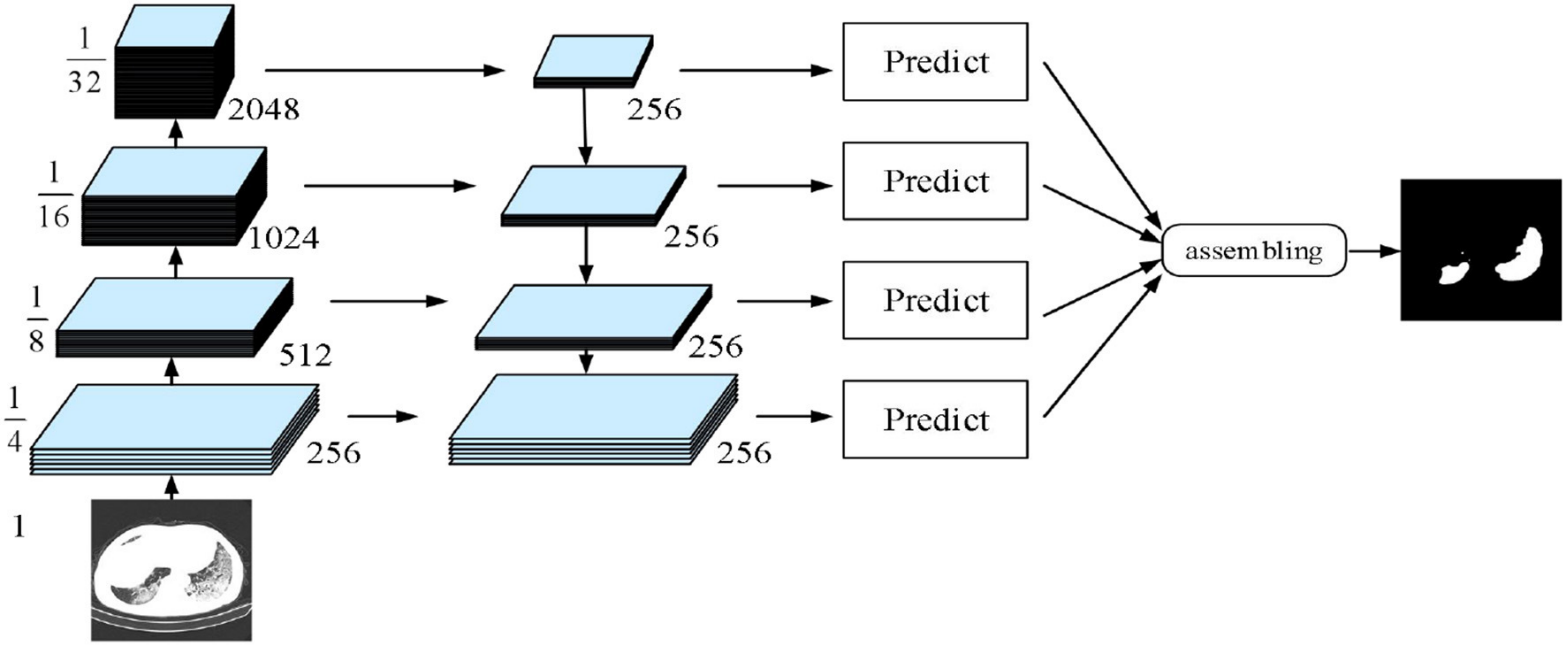
Feature pyramid network (FPN) model.

The fourth implemented architecture is the multi-scale feature pyramid network (FPN) by Lin et al. (44). As shown in Figure 7, a top-down architecture with skip connections was designed to express high-level feature maps at all scales. Subsequently, the semantic feature maps with different scales were integrated to improve the segmentation performance of COVID-19.

In these deep learning networks, training was performed using the Adam optimizer with a learning rate of 10^−5^, the dice loss was considered as the loss function to express the relationship between the predicted probabilities and the corresponding lesion annotations. Inspired by the previous work of Golla et al. (45), we present an ensemble module to ensemble the probability feature maps of four networks. To give a mathematical expression:


(8) $$\begin{array}{r} E=w_{1}\times E_{1}+w_{2}\times E_{2}+w_{3}\times E_{3}+w_{4}\times E_{4} \end{array}$$

Where *w*_1_, *w*_2_, *w*_3_, and *w*_4_ are the weighting parameters, *E*_1_, *E*_2_, *E*_3_, and *E*_4_ represent the predicted probabilities of PAN, FPN, Unet, and Deeplabv3+ networks. In which, the relationship among the weighting parameters *w*_1_, *w*_2_, *w*_3_, and *w*_4_ can be represented as follows:


(9) $$\begin{array}{r} w_{1}+w_{2}+w_{3}+w_{4}=1 \end{array}$$

To acquire the most effective segmentation results of COVID-19 lesions in CT images, an enumeration grid model (46) is used to achieve the optimal weighting parameters.

Weighting parameters play an important role in COVID-19 lesion segmentation. To overcome the problem, a simple but effective approach is designed to acquire the optimal weighting parameters with the enumeration grid model, which is a traversal method, it has the ability to enumerate all the parameters *w*_1_, *w*_2_, *w*_3_, and *w*_4_. First, *w*_1_ is set to be a fixed value varied from 0.0 to 1.0, and *w*_2_ and *w*_3_ are treated as variable values. As we all know that the sum of all the weighting parameters is 1.0, the weighting parameter *w*_4_ is 1–*w*_2_–*w*_3_. In other words, *w*_4_ is defined by variable *w*_2_and *w*_3_. As a result, all the weighting parameters and the corresponding quantitative index IOU can be acquired with the enumeration grid model. Since the IoU value difference is very small, it is difficult to distinguish. To better distinguish the quantitative index IoU for a good presentation, a simple mathematical transformation is given as:


(10) $$\begin{array}{r} Z=\log_{9/10}^{\left|IoU-\max\left(IoU\right)-0.0001\right|} \end{array}$$

**Figure 8:**
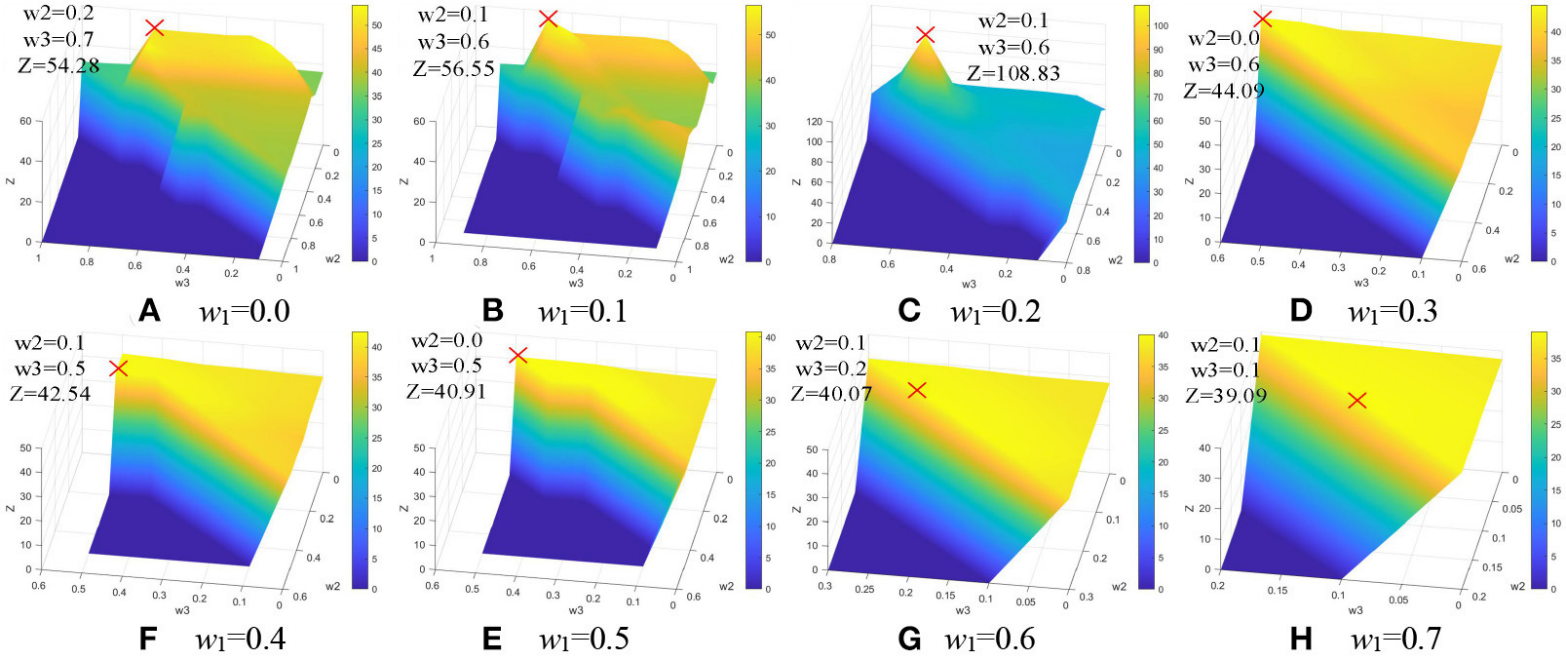
Weighting parameters optimization. **(A)** *w*_1_ = 0.0. **(B)** *w*_1_ = 0.1. **(C)** *w*_1_ = 0.2. **(D)** *w*_1_ = 0.3. **(E)** *w*_1_ = 0.4. **(F)** *w*_1_ =0.5. **(G)** *w*_1_ = 0.6. **(H)** *w*_1_ = 0.7.

As shown in Figure 8, the maximum values of Z are marked with a red cross and the corresponding values are described in Table 2. The optimal weighting parameters are 0.2, 0.1, 0.6, and 0.1.

**Table 2:** The maximum values of Z.

**Maximum points**				**Maximum values**
		*w*_3_	*w*_4_	Z
0.0	0.2	0.7	0.1	54.28
0.1	0.1	0.6	0.2	56.55
0.2	0.1	0.6	0.1	**108.83**
0.3	0.0	0.6	0.1	44.09
0.4	0.1	0.5	0	42.54
0.5	0.0	0.5	0	40.91
0.6	0.1	0.2	0.1	40.07
0.7	0.1	0.1	0.1	39.09
0.8	0	0.1	0.1	37.40
0.9	0	0	0.1	35.81
1.0	0	0	0	35.08

*Z = 108.83, is the largest values. It illustrates that the proposed method has the best segmentation performance in this case*.

Table 3 lists the architecture parameters with different networks. The dice loss function was regarded as the loss function. Adaptive moment estimation (Adam) was employed for the training process, which iteratively updates different network weights based on a publicly available novel coronavirus pneumonia dataset. The learning rate was initialized with 10^−5^. The above deep learning model was implemented in python using PyTorch with Lenovo Ren-9000 34IMZ, GPU GFX 2060, and CPU 32G.

**Table 3:** Architecture parameters with different networks.

**Parameters**	**Value**
Input image size	512 × 512
Output image size	512 × 512
Learning rate	10^−5^
Activation layers	Adam
Epochs	300
Batch size	4
Loss function	Dice

## Results

We present a deep-supervised ensemble learning network as an alternative model to segment COVID-19 lesions in CT images. The segmentation performance of the proposed method is validated in experiments with a publicly available dataset. The effectiveness of the proposed method was verified by visual inspection and quantitative evaluation.

### Visual Inspection

**Figure 9:**
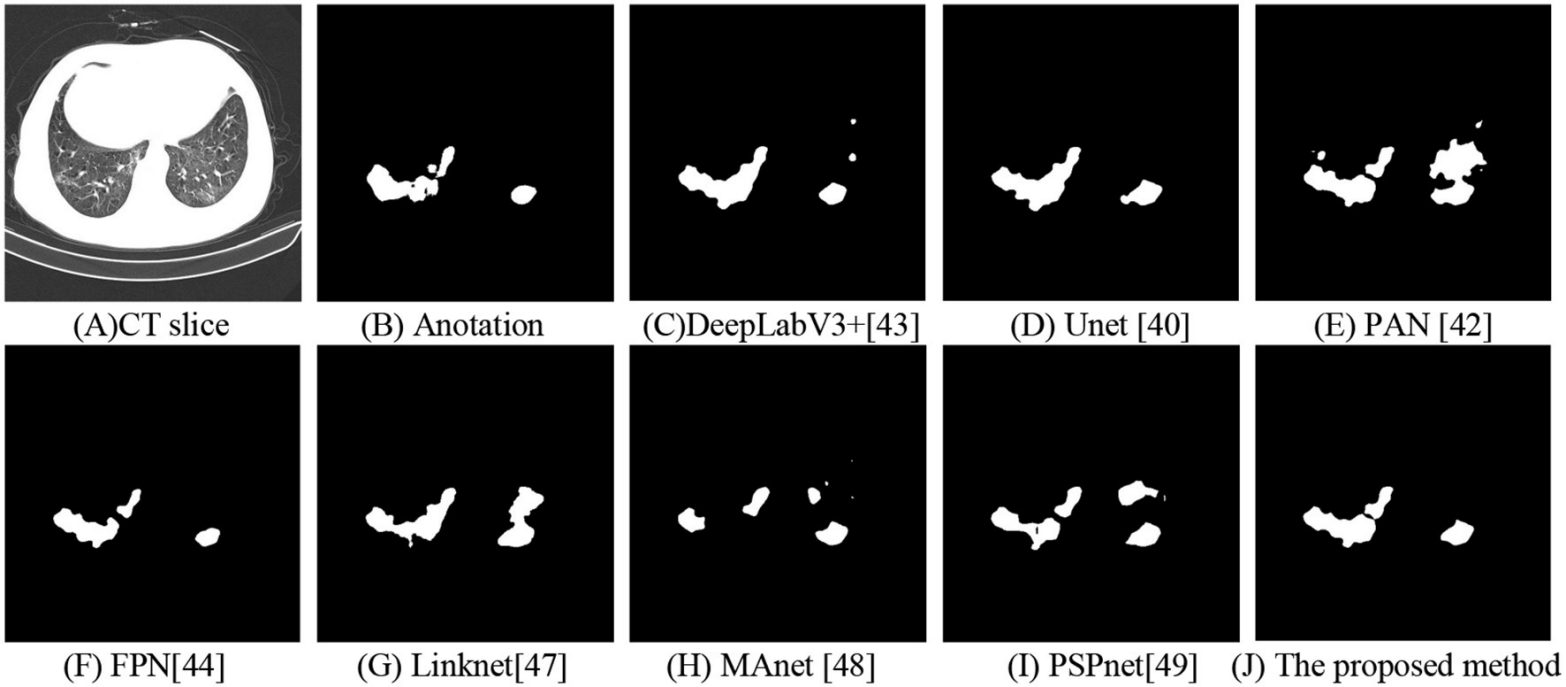
Segmentation of COVID-19 lesions with different deep learning methods. **(A)** CT slice. **(B)** Anotation. **(C)** DeepLabV3+. **(D)** Unet. **(E)** PAN. **(F)** FPN. **(G)** Linknet. **(H)** MAnet. **(I)** PSPnet. **(J)** The proposed method.

For visual inspection, we selected a typical CT slice from a public dataset for demonstration. The CT slice, the corresponding annotation, DeepLabV3+ (43), UNet (40), PAN (42), FPN (44), Linknet (47), MAnet (48), PSPnet (49), and the proposed method are displayed in Figure 9. In this, many methods (40, 43, 48, 49) used the local features to segment COVID-19 lesions, the approaches may cause parts of small lesions to be undetected. While parts of methods (42, 44, 47) exploited the local and global features for COVID-19 lesion segmentation, the strategy may cause parts of clutters to be unremoved. On the contrary, a deep-supervised ensemble learning network is presented to combine with the advantages of different deep learning networks (40, 42–44) for COVID-19 lesion segmentation. As observed, the proposed method has largely improved the segmentation results compared with seven deep learning networks (40, 42–44, 47–49). In other words, by using the deep-supervised ensemble learning network, the proposed method has a good performance in COVID-19 lesion segmentation.

**Figure 10:**
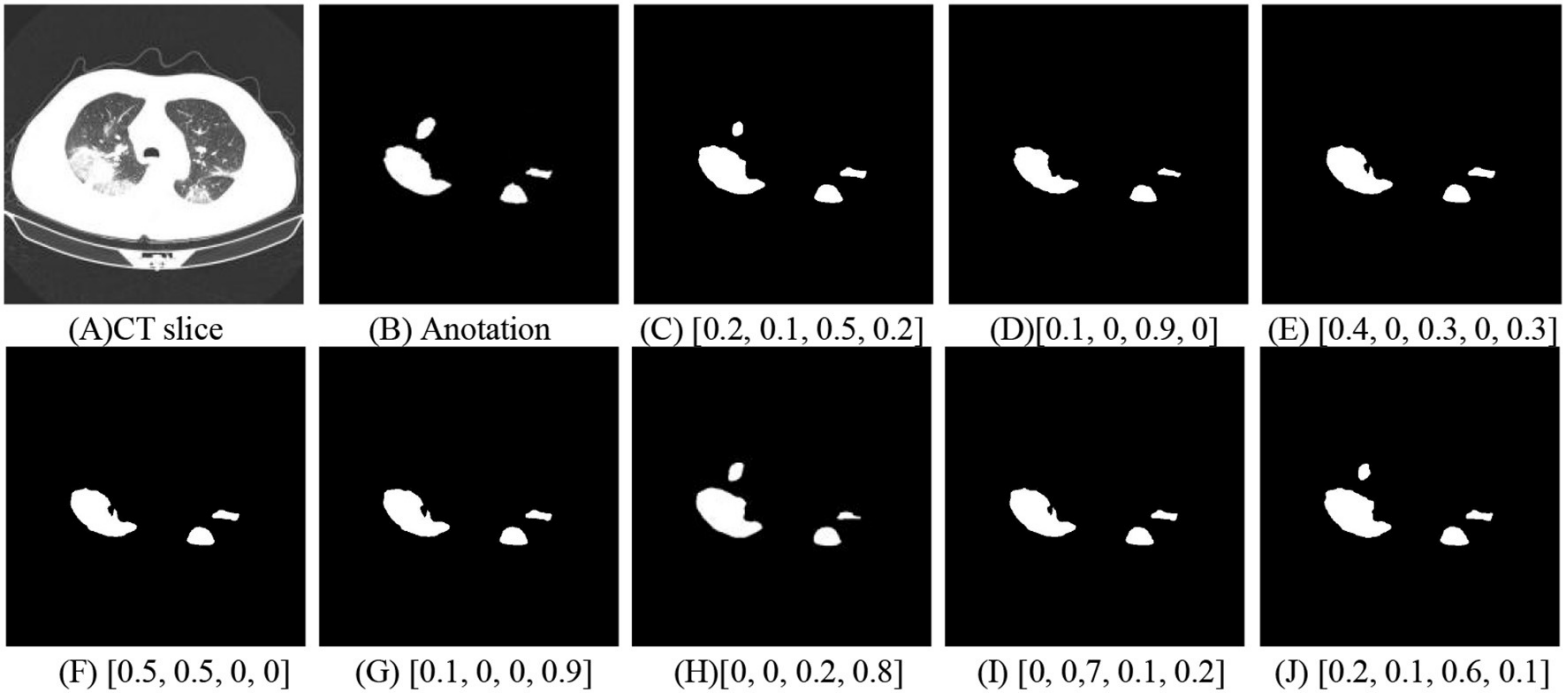
COVID-19 lesion segmentation with different weighting parameters. **(A)** CT slice. **(B)** Anotation. **(C)** [0.2, 0.1, 0.5, 0.2]. **(D)** [0.1, 0, 0.9, 0]. **(E)** [0.4, 0, 0.3, 0, 0.3]. **(F)** [0.5, 0.5, 0, 0]. **(G)** [0.1, 0, 0, 0.9]. **(H)** [0, 0, 0.2, 0.8]. **(I)** [0, 0.7, 0.1, 0.2]. **(J)** [0.2, 0.1, 0.6, 0.1].

To accurately segment novel coronavirus pneumonia lesions, the enumeration method is applied to estimate the best weighting parameters *w*_1_, *w*_2_, *w*_3_, and *w*_4_. In Figure 10, COVID-19 lesion segmentation results with different weighting parameters are displayed to evaluate the validation of the proposed method. It can be seen that the best weighting parameters are 0.2, 0.1, 0.6, and 0.1.

**Figure 11:**
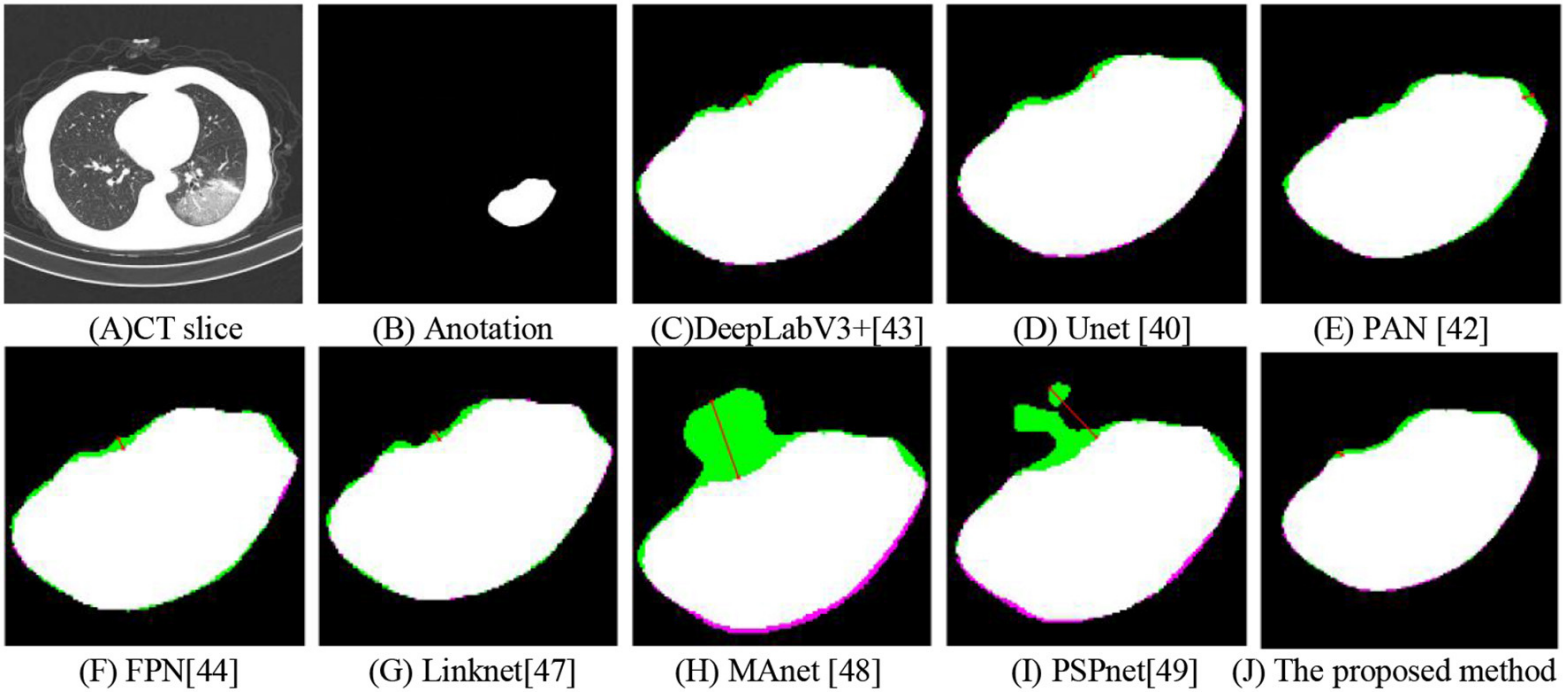
Hausdorff distance with different methods. **(A)** CT slice. **(B)** Anotation. **(C)** DeepLabV3+. **(D)** Unet. **(E)** PAN. **(F)** FPN. **(G)** Linknet. **(H)** MAnet. **(I)** PSPnet. **(J)** The proposed method.

To investigate the effect of the proposed method in COVID-19 lesion segmentation, a CT slice is chosen in Figure 11A and the corresponding annotation data are shown in Figure 11B. To better illustrate the effectiveness of the proposed method, the detected COVID-19 lesions with different methods were magnified. As shown in Figure 11, the pink region is the annotations, the green region is the predictions, and the white area is the common region covered by both predictions and annotations. In Figures 11C–J, the Hausdorff distance with different methods are 3.6056, 3.1623, 4.1231, 4.4721, 3.6056, 32.8938, 27.5862, and 2.2361. In other words, the proposed method has a lower Hausdorff distance than these typical methods (40, 42–44, 47–49).

### Quantitative Evaluation

We employed a dataset from the China consortium of chest CT image investigation, the dataset includes 750 CT images. As observed in Table 4, The IoU values corresponding to the DeepLabV3+ (43), Unet (40), PAN (42), FPN (44), Linknet (47), MAnet (48), PSPnet (49), and the proposed method are 0.7058, 0.6927, 0.7031, 0.7081, 0.6883, 0.6067, 0.6696, and 0.7279. Whereas the *F*_1_ values corresponding to different methods are 0.7886, 0.7720, 0.7881, 0.7931, 0.7618, 0.7216, 0.7557, and 0.8065, and the Hausdorff distance with different methods are 99.8686, 96.9404, 97.6020, 98.9161, 101.9168, 112.6191, 104.5176, and 92.4604. Both visual inspection and quantitative evaluation exhibited that our method can outperform these typical methods (40, 42–44, 47–49).

**Table 4:** The IoU values with different methods.

**Method**	**IoU**	**F_**1**_**	**H**
DeepLabV3+ (43)	0.7058	0.7886	99.8686
Unet (40)	0.6927	0.7720	96.9404
PAN (42)	0.7081	0.7931	97.6020
FPN (44)	0.7031	0.7881	98.9161
Linknet (47)	0.6883	0.7618	101.9168
MAnet (48)	0.6067	0.7216	112.6191
PSPnet (49)	0.6696	0.7557	104.5176
The proposed method	**0.7279**	**0.8065**	**92.4604**

*Compared with seven methods, the proposed method has the largest IoU value 0.7279 and F1 value 0.8065, which means that the proposed method has the best performance in regional sensitivity of COVID-19 lesion segmentation. What's more, the proposed method has the smallest H value 92.4604, which indicates that the computational model has the best performance in COVID-19 lesion boundary*.

The relationship between the weighting parameters and the IoU index is shown in Table 5. Both visual inspection and quantitative evaluation exhibited that the best weighting parameters are 0.2, 0.1, 0.6, and 0.1.

**Table 5:** The relationship between the weighting parameters and the IoU index.

***w*_1_**	***w*_2_**	***w*_3_**	***w*_4_**	**IoU**
0.1	0.0	0.9	0.0	0.6929
0.4	0.3	0.0	0.3	0.7083
0.0	0.7	0.1	0.2	0.7101
0.4	0.3	0.1	0.2	0.7108
0.7	0.1	0.1	0.1	0.7116
0.1	0.1	0.2	0.6	0.7232
0.2	0.3	0.4	0.1	0.7219
0.3	0.0	0.1	0.6	0.7150
0.5	0.1	0.2	0.2	0.7142
0.6	0.0	0.2	0.2	0.7120
0.8	0.0	0.0	0.2	0.7075
0.0	0.1	0.8	0.1	0.6951
0.2	0.0	0.7	0.1	0.7252
0.0	0.1	0.6	0.3	0.7229
0.2	0.1	0.6	0.1	**0.7279**

*Compared with different weighting parameters, the best weighting parameters are 0.2, 0.1, 0.6, and 0.1 and the maximum IoU value is 0.7279*.

## Discussion

In this article, a deep-supervised ensemble learning network is presented for COVID-19 segmentation in CT images, the proposed method has many specific characteristics and advantages. Based on the fact that a large number of COVID-19 CT images and the corresponding lesion annotations are difficult to be obtained, a transfer learning strategy is employed to make up for the shortcoming and alleviate the overfitting problem. Second, a unique ensemble module is presented to improve the segmentation performance. While many studies use only one neural network to segment COVID-19 lesions, which cannot effectively discriminate COVID-19 lesions and other unrelated structures in CT images. Third, the proposed method is expected to preserve the completeness of COVID-19 lesions while maximally eliminating the unrelated structures. Last, the proposed method has a good performance in COVID-19 lesion segmentation in CT images.

The proposed method was validated in a publicly available dataset from the China consortium of chest CT image investigation. Both visual inspection and quantitative evaluation exhibited that the proposed approach could outperform these typical deep learning networks in COVID-19 lesion segmentation (40, 42–44, 47–49). Compared with manually defined annotations, our methods obtained a higher accuracy in COVID-19 lesion segmentation with an IoU index of 0.7279 and an *F*_1_ value of 0.8065 than these typical methods. In which, many typical methods (40, 43, 48, 49) used the local features to segment COVID-19 lesions, the approaches may cause parts of small lesions to be undetected. While parts of methods (42, 44, 47) exploited the local and global features for COVID-19 lesion segmentation, the strategy may cause parts of clutters to be unremoved. On the contrary, a deep-supervised ensemble learning network is presented to combine with the advantages of different deep learning networks (40, 42–44) for COVID-19 lesion segmentation. In other words, the proposed method uses weight parameters to measure the importance of local and global features for COVID-19 lesion segmentation. While the compared methods used only one neural network to segment COVID-19 lesions, it may cause parts of COVID-19 lesions to be undetected.

Compared with these conventional neural networks (40, 42–44, 47–49), the proposed method appears more efficient on COVID-19 lesion segmentation. This is ascribed to a well-designed fusion of transfer learning strategy, data augmentation, and multiple neural networks-ensemble approaches. In other words, the proposed method outperforms the conventional methods in that the merits of local and global features are efficiently combined. In addition, the segmentation of COVID-19 lesions has important clinical research significance. It can help doctors to diagnosis COVID-19 and develop the best treatment plan.

However, the designed deep-supervised ensemble learning network may take up more time and space than traditional conventional neural networks (40, 42–44, 47–49). Additionally, based on the fact that a large number of COVID-19 CT images and the corresponding lesion annotations are difficult to be obtained, accurate segmentation of small coronavirus pneumonia lesions is still a long way off.

In conclusion, a deep-supervised ensemble learning network is presented for coronavirus pneumonia lesion segmentation in CT images. Based on the reality that mounts of COVID-19 CT images and the corresponding lesion annotations are difficult to acquire, a transfer learning strategy is used to alleviate the overfitting problem in a small dataset. Another contribution of the proposed method concerns the deep-supervised ensemble learning network. Using a single deep learning network, the accuracy of COVID-19 lesion segmentation results cannot reach a satisfactory performance. To overcome the problem, an ensemble strategy is presented to integrate multiple deep learning networks for COVID-19 lesion and its boundary segmentation in CT images. Experimental results indicated that our proposed deep-supervised ensemble learning model has a good performance in COVID-19 lesion and its boundary segmentation in CT images.

## Data Availability Statement

The original contributions presented in the study are included in the article/supplementary material, further inquiries can be directed to the corresponding author/s.

## Author Contributions

YP was the major contributor in writing the manuscript. All authors analyzed the data, contributed to the article, and approved the submitted version.

## Funding

This research was funded by the Jiangxi Provincial Natural Science Foundation (Nos. 20212BAB202007, 20202BAB212004, 20212BAB211009, 20204BCJL23035, 20192ACB21004, and 20181BAB202017), by the Educational Science Research Project of China Institute of communications Education (No. JTYB20-33), by the Huxiang high-level talent gathering project (No. 2019RS1072), by the Scientific and Technological Research Project of Education Department in Jiangxi Province (No. GJJ190356), and the Science and Technology project of Changsha City (No. kq2001014).

## Conflict of Interest

HT was employed by company Technique Center, Hunan Great Wall Technology Information Co. Ltd. The remaining authors declare that the research was conducted in the absence of any commercial or financial relationships that could be construed as a potential conflict of interest.

## Publisher's Note

All claims expressed in this article are solely those of the authors and do not necessarily represent those of their affiliated organizations, or those of the publisher, the editors and the reviewers. Any product that may be evaluated in this article, or claim that may be made by its manufacturer, is not guaranteed or endorsed by the publisher.

## References

[1] Alon D Paitan Y Robinson E Ganor N Lipovetsky J Yerushalmi R. Downregulation of CD45 Signaling in COVID-19 patients is reversed by C24D, a novel CD45 targeting peptide. Front Med. (2021) 8:1251. 10.3389/fmed.2021.675963 34414199 PMC8369232

[2] Murphy A Pinkerton LM Bruckner E Risser HJ. The impact of the novel coronavirus disease 2019 on therapy service delivery for children with disabilities. J Pediatr. (2021) 231:168–77. 10.1016/j.jpeds.2020.12.060 33359629 PMC7982784

[3] Comunale BA Engineer L Jiang Y Andrews JC Liu Q Ji L. Poliovirus vaccination induces a humoral immune response that cross reacts with SARS-CoV-2. Front Med. (2021) 8:1285. 10.3389/fmed.2021.710010 34414206 PMC8369257

[4] Li L Cao Y Fan J Li T Lang J Zhang H. Impact of COVID-19 pandemic on the clinical activities in obstetrics and gynecology: a national survey in China. Front Med. (2021) 8:1225. 10.3389/fmed.2021.633477 34395457 PMC8360866

[5] Meng Z Wang T Chen L Chen X Li L Qin X. The effect of recombinant human interferon alpha nasal drops to prevent COVID-19 pneumonia for medical staff in an epidemic area. Curr Top Med Chem. (2021) 21:920–7. 10.2174/1568026621666210429083050 33970846

[6] Herpe G Lederlin M Naudin M Obana M Chaumoitre K Gregory J. Efficacy of chest CT for COVID-19 pneumonia diagnosis in France. Radiology. (2021) 298:81–7. 10.1148/radiol.2020202568 32870139 PMC7465292

[7] Zhao Y Ji D Li Y Zhao X Lv W Xin X. Three-dimensional visualization of microvasculature from few-projection data using a novel CT reconstruction algorithm for propagation-based X-ray phase-contrast imaging. Biomed Opt Express. (2020) 11:364–87. 10.1364/BOE.380084 32010522 PMC6968748

[8] Schalekarmp S Bleeker-Rovers CP Beenen LFM Quarles van Ufford HME Gietema HA Stoger JL. Chest CT in the emergency department for diagnosis of COVID-19 pneumonia: dutch experience. Radiology. (2021) 298:98–106. 10.1148/radiol.2020203465 33201791 PMC7676748

[9] Bhargava A Bansal A. Novel coronavirus (COVID-19) diagnosis using computer vision and artificial intelligence techniques: a review. Multimed Tools Appl. (2021) 80:19931–46. 10.1007/s11042-021-10714-5 33686333 PMC7928188

[10] Peng Y Xiao C. An oriented derivative of stick filter and post-processing segmentation algorithms for pulmonary fissure detection in CT images. Biomed Signal Proces. (2018) 43:278–88. 10.1016/j.bspc.2018.03.013

[11] Peng Y Zhong H Xu Z Tu H Li X Peng L. Pulmonary lobe segmentation in CT images based on lung anatomy knowledge. Math Probl Eng. (2021) 2021:5588629. 10.1155/2021/5588629

[12] Zhang Y Hu Y Zhao S Cui C. The utility of PET/CT metabolic parameters measured based on fixed percentage threshold of SUVmax and adaptive iterative algorithm in the new revised FIGO staging system for stage III cervical cancer. Front Med. (2021) 8:1189. 10.3389/fmed.2021.680072 34395472 PMC8358139

[13] Chen S Chen J Yang Y Chien C Wang M Lin L. Use of radiographic features in COVID-19 diagnosis: challenges and perspectives. J Chin Med Assoc. (2020) 83:644–7. 10.1097/JCMA.0000000000000336 32349032 PMC7434022

[14] Dastidar TR Ethirajan R. Whole slide imaging system using deep learning-based automated focusing. Biomed Opt Express. (2020) 11:480–91. 10.1364/BOE.379780 32010529 PMC6968754

[15] Ali MJ Hanif M Haider MA Ahmed MU Sundas F Hirani A. Treatment options for COVID-19: a review. Front Med. (2020) 7:480. 10.3389/fmed.2020.00480 32850922 PMC7412857

[16] Hammoudi K Benhabiles H Melkemi M Dornaika F Arganda-Carreras I Collard D. Deep learning on chest X-ray images to detect and evaluate pneumonia cases at the ear of COVID-19. J Med Syst. (2021) 45:75. 10.1007/s10916-021-01745-4 34101042 PMC8185498

[17] Erion G Janizek JD Sturmfels P Lundberg SM Lee S. Improving performance of deep learning models with axiomatic attribution priors and expected gradients. Nat Mach Intell. (2021) 37:1–12. 10.1038/s42256-021-00343-w

[18] Yazdekhasty P Zindar A Nabizadeh-ShahreBabak Z Roshandel R Khadivi P Karimi N. Bifurcated autoencoder for segmentation of COVID-19 infected regions in CT images. arXiv preprint 2011. (2020) p. 00631. 10.1007/978-3-030-68790-8_46

[19] Wang Y Zhang Y Liu Y Tian J Zhong C Shi Z. Does non-COVID-19 lung lesion help? Investigating transferability in COVID-19 CT image segmentation. Comput Meth Prog Bio. (2021) 202:106004. 10.1016/j.cmpb.2021.106004 33662804 PMC7899930

[20] Gao K Su J Jiang Z Zeng L Feng Z Shen H. Dual-branch combination network (DCN): Towards accurate diagnosis and lesion segmentation of COVID-19 using CT images. Med Image Anal. (2021) 67:101836. 10.1016/j.media.2020.101836 33129141 PMC7543739

[21] Zhao S Li Z Chen Y Zhao W Xie X Liu J. SCOAT-Net: A novel network for segmentation COVID-19 lung opacification from CT images. Pattern Recogn. (2021) 119:108109. 10.1016/j.patcog.2021.108109 34127870 PMC8189738

[22] Abdel-Basset M Chang V Hawash H Chakrabortty RK Ryan M. FSS-2019-nCov: a deep learning architecture for semi-supervised few-shot segmentation of COVID-19 infection. Knowl-Based Syst. (2021) 212:106647. 10.1016/j.knosys.2020.106647 33519100 PMC7836902

[23] Yang D Xu Z Li W Myronenko A Roth HR Harmon S. Federated semi-supervised learning for COVID region segmentation in Chest CT using multi-national data from China, Italy, Japan. Med Image Anal. (2021) 70:101992. 10.1016/j.media.2021.101992 33601166 PMC7864789

[24] Piccolo V Neri I Filippeschi C Oranges T Argenziano G Battarra VC. Chilblain-like during COVID-19 epidemic: a preliminary study on 63 patients. J Eur Acad Dermatol. (2020) 34:e291–e345. 10.1111/jdv.16526 32330334 PMC7267498

[25] Yang Y Chen J Wang R Ma T Wang L Chen J. Towards unbiased COVID-19 lesion localisation and segmentation via weakly supervised learning. In: IEEE 18th International Symposium on Biomedical Imaging. (2021). p. 1966–70. 10.1109/ISBI48211.2021.943380627295638

[26] Laradji I Rodriguez P Manas O Lensink K Law M Kurzman L. A weakly supervised consistency-based learning method for COVID-19 segmentation in CT images. In: IEEE Winter Conference on Applications of Computer Vision. (2021). p. 2453–62. 10.1109/WACV48630.2021.0025027295638

[27] Wu X Chen C Zhong M Wang J Shi J. COVID-AL the diagnosis of COVID-19 with deep active learning. Med Image Anal. (2021) 68:101913. 10.1016/j.media.2020.101913 33285482 PMC7689310

[28] Wang X Deng X Fu Q Zhou Q Feng J Ma H. A weakly supervised framework for COVID-19 classification and lesion localization from chest CT. IEEE Trans Med Imaging. (2020) 39:2615–25. 10.1109/TMI.2020.2995965 33156775

[29] Yao Q Xiao L Liu P Zhou SK. Label-free segmentation of COVID-19 lesions in lung CT. IEEE Trans Med Imaging. (2021) 40:2808–19. 10.1109/TMI.2021.3066161 33760731 PMC8544940

[30] Zhang K Liu X Shen J Li Z Sang Ye Wu X. Clinically applicable AI system for accurate diagnosis, quantitative measurements, and prognosis of COVID-19 pneumonia using computed tomography. Cell. (2020) 181:1423–33. 10.1016/j.cell.2020.04.045 32416069 PMC7196900

[31] Zhu Y Yeung CH Lam EY. Digital holographic imaging and classification of microplastics using deep transfer learning. Appl Optics. (2021) 60:38–47. 10.1364/AO.403366 33690352

[32] Fu Y Lei Y Wang T Curran WJ Liu T Yang X. Deep learning in medical image registration: a review. Phys Med Biol. (2020) 65:20TR01. 10.1088/1361-6560/ab843e 32217829 PMC7759388

[33] Du S Du J Tang Y Ouyang H Tao Z Jiang T. Achieving efficient inverse design of low-dimensional heterostructures based on a vigorous scalable multi-task learning network. Opt Express. (2021) 29:19727–42. 10.1364/OE.426968 34266077

[34] Christensen CN Ward EN Lu M Lio P Kaminski CF. ML-SIM universal reconstruction of structured illumination microscopy images using transfer learning. Biomed Opt Express. (2021) 12:2720–33. 10.1364/BOE.414680 34123499 PMC8176814

[35] Wang SH Fernandes S Zhu Z Zhang YD. AVNC: attention-based VGG-style network for COVID-19 diagnosis by CBAM. IEEE Sens J. (2021). 10.1109/JSEN.2021.3062442 36346097 PMC9564036

[36] Krizhevsky A Sutskever I Hinton GE. ImageNet classification with deep convolutional neural networks. Commun Acm. (2017) 60:84–90. 10.1145/3065386

[37] Marques G Agarwal D Diez IDLT. Automated medical diagnosis of COVID-19 through EfficientNet convolutional neural network. Appl Soft Comput. (2020) 96:106691. 10.1016/j.asoc.2020.106691 33519327 PMC7836808

[38] Munien C Viriri S. Classification of hematoxylin and eosinstained breast cancer histology microcopy images using transfer learning with EfficientNets. Comput Intel Neurosc. (2021) 2021:5580914. 10.1155/2021/5580914 33897774 PMC8052174

[39] Xie Q Luong M Hovy E Le QV. Self-training with noisy student improves ImageNet classification. In: CVPR. (2020) 10.1109/CVPR42600.2020.0107027295638

[40] Su R Zhang D Liu J Cheng C. MSU-Net: Multi-scale U-Net for 2D medical image segmentation. Front Genet. (2021) 12:140. 10.3389/fgene.2021.639930 33679900 PMC7928319

[41] Wang B Yang J Ai J Luo N An L Feng H. Accurate tumor segmentation via octave convolution neural network. Frontiers in Medicine. (2021) 8:653913. 10.3389/fmed.2021.653913 34095168 PMC8169966

[42] Li H Xiong P An J Wang L. Pyramid attention network for semantic segmentation. arXiv preprint (2018). p. 1805.

[43] Chen L C Zhu Y Papandreou G Schroff F Adam H. Encoder-decoder with atrous separable convolution for semantic image segmentation. In: ECCV, (2018). p. 801–18. 10.1007/978-3-030-01234-2_49

[44] Lin TY Dollar P Girshick R He K Hariharan B Belongie S. Feature pyramid networks for object detection. In: CVPR, (2017). p. 2117–25. 10.1109/CVPR.2017.10627295638

[45] Golla AK Bauer DF Schmidt R Russ T Norenberg D Chung K. Convolutional neural network ensemble segmentation with ratio-based sampling for the arteries and veins in abdominal CT scans. IEEE Trans Bio-med Eng. (2021) 68:1518–26. 10.1109/TBME.2020.3042640 33275574

[46] Hartley MG Ralph E. Norville lH, Prior JL, Atkins TP. Comparison of PCR and viable count as a method for enumeration of bacteria in an A/J mouse aerosol model of Q fever. Front Microbiol. (2019) 10:1552. 10.3389/fmicb.2019.01552 31379760 PMC6647910

[47] Chaurasia A Culurciello E. Linknet: exploiting encoder representations for efficient semantic segmentation. In: VCIP. (2017). p. 1–4. 10.1109/VCIP.2017.830514827295638

[48] Fan T Wang G Li Y Wang H. MA-Net: A Multi-Scale attention network for liver and tumor segmentation. IEEE Access. (2020) 8:179656–65. 10.1109/ACCESS.2020.302537227295638

[49] Zhao H Shi J Qi X Wang X Jia J. Pyramid scene parsing network. In: CVPR. (2017). p. 2881–90. 10.1109/CVPR.2017.66027295638

